# A Robotics Experimental Design Method Based on PDCA: A Case Study of Wall-Following Robots

**DOI:** 10.3390/s24061869

**Published:** 2024-03-14

**Authors:** Kai-Yi Wong, Shuai-Cheng Pu, Ching-Chang Wong

**Affiliations:** 1 Department of Electrical Engineering, National Sun Yat-sen University, Kaohsiung City 80424, Taiwan; kywong@mail.ee.nsysu.edu.tw; 2 Department of Electrical and Computer Engineering, Tamkang University, New Taipei City 25137, Taiwan; 807440044@gms.tku.edu.tw

**Keywords:** robotics experiment design, multi-sensor fusion, plan-do-check-act (PDCA), robot assembly and control, autonomous mobile robot

## Abstract

There is a lack of research that proposes a complete and interoperable robotics experimental design method to improve students’ learning outcomes. Therefore, this study proposes a student-oriented method based on the plan-do-check-act (PDCA) concept to design robotics experiments. The proposed method is based on our teaching experience and multiple practical experiences of allowing students to do hands-on experiments. It consists of eight steps, mainly including experimental goals, experimental activities, robot assembly, robot control, in-class evaluation criteria, and after-class report requirements. The after-class report requirements designed in the proposed method can help students improve their report-writing abilities. A wall-following robotics experiment designed using the PDCA method is proposed, and some students’ learning outcomes and after-class reports in this experiment are presented to illustrate the effectiveness of the proposed method. This experiment also helps students to understand the fundamental application of multi-sensor fusion technology in designing an autonomous mobile robot. We can see that the proposed reference examples allow students to quickly assemble two-wheeled mobile robots with four different sensors and to design programs to control these assembled robots. In addition, the proposed in-class evaluation criteria stimulate students’ creativity in assembling different wall-following robots or designing different programs to achieve this experiment. We present the learning outcomes of three stages of the wall-following robotics experiment. Three groups of 42, 37, and 44 students participated in the experiment in these three stages, respectively. The ratios of the time required for the robots designed by students to complete the wall-following experiment, less than that of the teaching example, are 3/42 = 7.14%, 26/37 = 70.27%, and 44/44 = 100%, respectively. From the comparison of learning outcomes in the three stages, it can be seen that the proposed PDCA-based design method can indeed improve students’ learning outcomes and stimulate their active learning and creativity.

## 1. Introduction

In the digital age, the importance of robotics education is increasingly valued [1,2]. Robotics education can foster students’ interest in digital computing [3]. Furthermore, dynamic learning through robots will have a positive impact on students [4]. The core disciplines of robotics encompass fundamental sciences, physics, mathematics, computer science, mechanical engineering, electrical engineering, and automation to fulfill various functionalities required by humans [5]. Mobile robots are one of the platforms that are often used for robotics education [6]. Related discussions include multi-sensor fusion [7], localization [8], navigation [9], and simultaneous localization and mapping [10]. Meanwhile, many educational institutions are developing practical robotics experiments. The commonly used software and hardware in various works in the robotics course-related literature are shown in Table 1. LEGO robotics kits with graphical programming languages are commonly used educational resources in the field of robotics. Compared with traditional teaching methods, they have a more positive impact on improving students’ problem-solving abilities and generating learning motivation [11,12,13].

A review of this literature can provide insight into teaching trends and some details of the courses. However, most works in the literature focus on the course content, and few focus on the design methods of robotics experiments and students’ hands-on experimental results. Here is a comparison of methods that can be applied to course design. DMAIC (Define–Measure–Analyze–Improve–Control) [26,27] is a data-oriented problem-solving method suitable for larger-scale projects. PDSA (Plan–Do–Study–Act) [28,29,30] is a modification of the original PDCA, which changes Check in the third step to Study (Study Assessment), which is suitable for longer-term projects. Compared with DMAIC and PDSA, the cycle design concept of PDCA is more suitable for the student-learning-outcome-oriented design of the robotics experimental course in this study. PDCA is a quality management model based on the initial PDCA process proposed by Edwards Deming [31]. It consists of the following phases: (1) Plan (P): Plan a solution method for the discovered problem. (2) Do (D): Execute the planned solution method. (3) Check (C): Check the execution results of the planned method. (4) Action (A): Improve the planned method. Based on the PDCA cycle adjustment of this quality management method, better solutions and results can be obtained [32]. Moreover, integrating the PDCA method into course design can improve the quality of course development [33]. When applied to teaching methods, PDCA is considered to be effective in providing beginners with a systematic step-by-step approach to solve problems. It saves students’ learning time and enhances their ability to learn to use different software [34]. Based on the PDCA concept, this study proposes eight steps to design robotics experiments. The structure is clear and simple, and these steps are tailored for hands-on robotics experiments. Moreover, the design of an experiment for wall-following robots with PDCA cycle steps is also used to illustrate the PDCA design method. The steps proposed in this study are derived from our improved process and teaching experience from some of our actual robotics experiments. The proposed method can be flexible and easily applied to the design of other fundamental robotics experiments. In addition, the proposed method will be applied to process other complex robots [35].

Furthermore, three requirements are often encountered when designing robotics experiments: (a) An experimental method and content that is interoperable across platforms is required [36]. Many types of software and hardware of robot platforms used in experiments are incompatible, so it is challenging to obtain relevant experimental design methods and teaching experience from different types of robot platforms. (b) Complete teaching resources are required [37,38]. Experiments not only require relevant teaching content, but also the development methods and processes of this teaching content and of student learning outcomes. (c) Students’ learning outcomes are required to be considered [33]. The design of the experiment needs to come from the students’ perspective, so that students can quickly understand the purpose and methods of the experiment, which can stimulate students’ creativity and learning motivation. Therefore, this study proposes corresponding solutions to the above three requirements: (a) We provide experimental content that is interoperable across platforms. Although the teaching case of this study uses the LEGO-EV3 kit and the graphical programming language EV3-G, the proposed PDCA-based robotics experimental design method and teaching experience can be easily applied to other robot platforms (Arduino, Raspberry Pi, etc.) and programming languages (C, C++, MATLAB, Python, etc.). (b) We propose a PDCA-based robotics experimental design method, and take a wall-following robot as an example to introduce in detail the design process and results of the design method. In addition, the effectiveness of this design method is demonstrated through the development process of the teaching content and student learning outcomes through repeated teaching. (c) We provide some step-by-step robotics experiment design examples. Based on the scaffolding theory [39,40], we provide some reference examples of robot assembly and control for the wall-following robotics experiment. This guides students to extend and apply the reference examples, making it easier for them to effortlessly understand the goals of robotics experiments and the principal key concepts in robot assembly and control. By summarizing students’ learning outcomes, it is evident that they can use the PDCA concept to assemble robots or design control programs which are not only creative but also perform better than the reference examples. Therefore, there are three main contributions.

(i) The proposed experimental design method has teaching scalability. Although the robot kit LEGO EV3 and the graphical programming language EV3-G are used in this design, and only the case of a wall-following robot is described, the proposed method can be extended and applied to different robot platforms and cases. Teachers in related fields can easily prepare and design other robotics experiments on their robot platforms based on the proposed method.
(ii) An actual teaching process and experience of the proposed PDCA method in the experimental design of wall-following robots are introduced, and the learning outcomes of students in the actual hands-on process and after-class reports are described. Teachers in related fields can refer to the proposed method to efficiently design other robotics experiments to improve the integrity of the teaching materials.
(iii) The proposed method is a student-oriented design method. The implemented experimental course not only allows students to achieve good learning results in robot assembly and control but also stimulates students’ active learning and creativity. It can be seen from the students’ learning outcomes in the third stage of the wall-following robotics experiment that the proposed reference examples allow students to quickly assemble robots and design programs to control the assembled robots. In addition, some students further stimulate their creativity to assemble different robots or control programs to achieve the experimental goal more quickly.

There are five sections in this study. In Section 1, the background is introduced. In Section 2, a PDCA-based robotics experimental design method is proposed. In Section 3, a wall-following robotics experiment is taken as a case to illustrate the eight steps of the proposed PDCA-based design method. In Section 4, some students’ learning outcomes and students’ creations in robot assembly and robot control are described. Finally, conclusions are described in Section 5.

## 2. PDCA-Based Robotics Experimental Design Method

**Figure 1:**
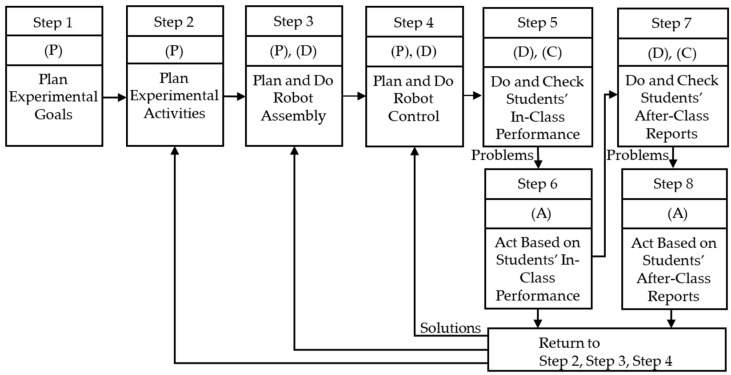
Design process of the proposed PDCA-based robotics experimental design method.

The design process of the proposed PDCA-based robotics experimental design method is shown in Figure 1. There are eight steps in total, and the specific instructions are as follows.

Step 1: Plan Experimental Goals

(Plan) Teachers plan which fundamental abilities students should have in robot assembly (fundamental assembly and application of mechanical structures and sensors) and robot control (fundamental design of control programs), and in other aspects.

Step 2: Plan Experimental Activities

(Plan) Teachers plan some experimental activities that allow students to assemble a robot and design a program to control this robot within a specified time and plan an experimental field. In addition, teachers plan some requirements for students’ in-class performance and after-class reports that can stimulate students’ active learning and creativity.

Step 3: Plan and Do Robot Assembly

(Plan) Teachers plan a teaching example of robot assembly so that students can complete the assembly of the mechanical structure and sensors of this robotics experiment, and students are willing to actively learn and use their creativity to assemble different robots to complete this experiment.
(Do) First, teachers use some mechanical components and sensors to design a test version of the robot assembly that can complete the planned experiment. Then, teachers repeatedly test the suitability of the planned robot assembly. If problems are found, teachers modify the way in which the robot is assembled. If there are no problems, a teaching example of the robot assembly will be generated.

Step 4: Plan and Do Robot Control

(Plan) Teachers plan a teaching example of robot control so that students can use some programming syntax structures to design the program of this robotics experiment, and students are willing to actively learn and use their creativity to design different programs to complete this experiment.
(Do) First, teachers use some programming syntax to design a test version of the control program that can complete the planned experiment. Then, teachers repeatedly test the suitability of the planned robot control. If problems are found, teachers modify the control program. If there are no problems, a teaching example of robot control will be generated.

Step 5: Do and Check Students’ In-Class Performance

(Do) Teachers perform the teaching examples completed in Steps 3 and 4 and allow students to conduct actual hands-on experiments with physical robots in the classroom.
(Check) Based on the in-class evaluation criteria planned in Step 2, teachers check the students’ learning outcomes in experimental activities, robot assembly, robot control, and in-class performance.

Step 6: Act Based on Students’ In-Class Performance

(Act) If the experimental activities, robot assembly, or robot control need improvement, teachers should return to Step 2, Step 3, or Step 4 to re-plan. For example, if some students cannot complete the experiment within the specified time, teachers should add some reference examples of robot assembly and robot control so that all students can complete the experiment within the specified time. On the other hand, if most students complete the experiment quickly, teachers should increase the difficulty of the experimental activities to allow students to obtain more learning results.

Step 7: Do and Check Students’ After-Class Reports

(Do) Teachers perform the correction and analysis of reports submitted by students.
(Check) Based on the requirements of the after-class report planned in Step 2, teachers check the students’ learning outcomes in experimental activities, robot assembly, robot control, and after-class report.

Step 8: Act Based on Students’ After-Class Reports

(Act) If the experimental activities, robot assembly, or robot control need improvement, teachers should return to Step 2, Step 3, or Step 4 to re-plan. For example, teachers should analyze whether the requirements for after-class reports make the content of the report more orderly, specific, and complete (improving students’ ability to write reports), and whether some requirements need to be added to further improve the results of after-class reports. If there are positive and negative suggestions, teachers should analyze and discuss whether relevant planning and teaching projects should be adjusted.

## 3. PDCA-Based Robotics Experimental Design for Wall-Following

A wall-following robotics experiment is taken as a case to illustrate the eight steps of the proposed PDCA-based design method described in Figure 1. With the LEGO EV3 kit and the graphical programming language EV3-G, most beginners can have the chance to learn robotics and programming easily and quickly. Therefore, this design uses EV3 and EV3-G to illustrate the proposed method.

Step 1: Plan Experimental Goals

(Plan) In the case of the wall-following robotics experiment, the main experimental goals are for students to possess two fundamental abilities after completing this experiment: (a) Students will be able to use some components and sensors to assemble a wall-following robot. (b) Students will be able to use some programming syntax to control the assembled robot. In addition, students can also develop the fundamental ability to write specific after-class reports through this experimental process.

**Figure 2:**
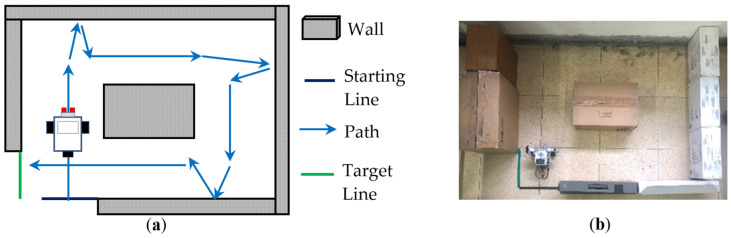
One-way wall-following experimental activities and experimental field planned at the beginning of the PDCA cyclic improvement process. (**a**) One-way moving sequence. (**b**) Photo of the experimental field.

**Figure 3:**
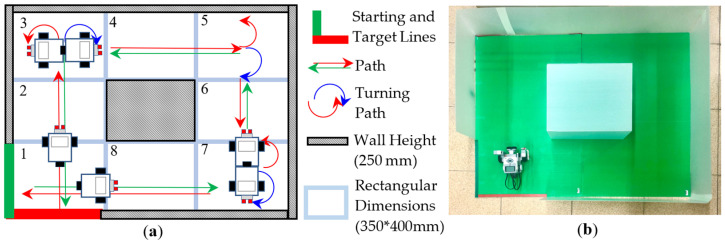
Two-way wall-following experimental activities and experimental field planned after the PDCA cyclic improvement process, (**a**) Two-way moving sequence. (**b**) Photo of the experimental field.

Step 2: Plan Experimental Activities

(Plan) Based on the experimental goals planned in Step 1, we initially planned some experimental activities and an experimental field for the wall-following robot, as shown in Figure 2. Its activity mode is that the robot must move clockwise along the wall from the starting line (the black line) to the target line (the green line). In addition, we planned to establish some teaching examples, so that the students could use the robot kit LEGO EV3 to assemble a two-wheeled robot and use the graphical programming language EV3-G to design a program to control this assembled robot within a specified time of three hours to complete this experiment. In the PDCA cyclic improvement process, in order to increase the difficulty of the experiment, the final improved experimental activities and an experimental field are shown in Figure 3. We modified the original activity mode from one-way (clockwise) wall-following movement to two-way movement (clockwise and counterclockwise); whether the moving sequence was clockwise (1 → 2 → 3 →…→ 8) or counterclockwise (1 → 8 → 7 →…→ 2), students had to assemble some sensors so that the robot could sense the distance between itself and the wall and determine a correct direction to turn at corners to complete the experiment. In the planning of the in-class evaluation criteria, we initially only provided rough evaluation criteria such as “unfinished”, “partially completed”, and “completed”. Therefore, some students may have problems in the classroom, such as unclear stage objectives or low motivation for active learning. For the PDCA cyclic improvement process, the final in-class evaluation criteria are shown in Table 2. Four levels (A, B, C, D) of the four completion modes enable students to gain a clearer understanding of the stage objectives in the in-class learning process and encourage students towards active learning. Furthermore, in an environment of peer tutoring and peer competition, students are more likely to be motivated to complete better or more creative wall-following robots. In the planning of the requirements of the after-class report, we initially only provided rough requirements of four topics, including “experimental objectives”, “experimental tasks and principles”, “experimental results”, and “learning experience and feedback”. Therefore, some students’ reports may have problems such as confusing content order or unclear text descriptions. In the PDCA cyclic improvement process, the final requirements of the after-class report are shown in Table 3. In these improved requirements, each topic has some sub-topics, and there are some clear requirements to prompt which specific content needs to be included in the report. This allows students to organize reports sequentially, according to the prescribed sub-topics. In addition, these improved requirements let students know that they must take photos and record some information in the classroom to make their reports specific and complete.

**Figure 4:**
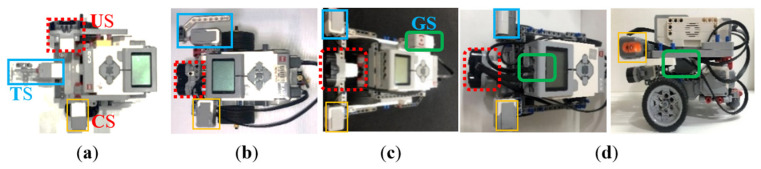
Multiple robots designed in the robot assembly process of the wall-following robotics experiment before and after the PDCA cyclic improvement process. (**a**) Robot with a touch sensor placed in the front. (**b**) Robot with an ultrasonic sensor placed in the front. (**c**) Robot with a gyro sensor not placed in the middle. (**d**) Robot with a gyro sensor placed in the middle.

**Figure 5:**
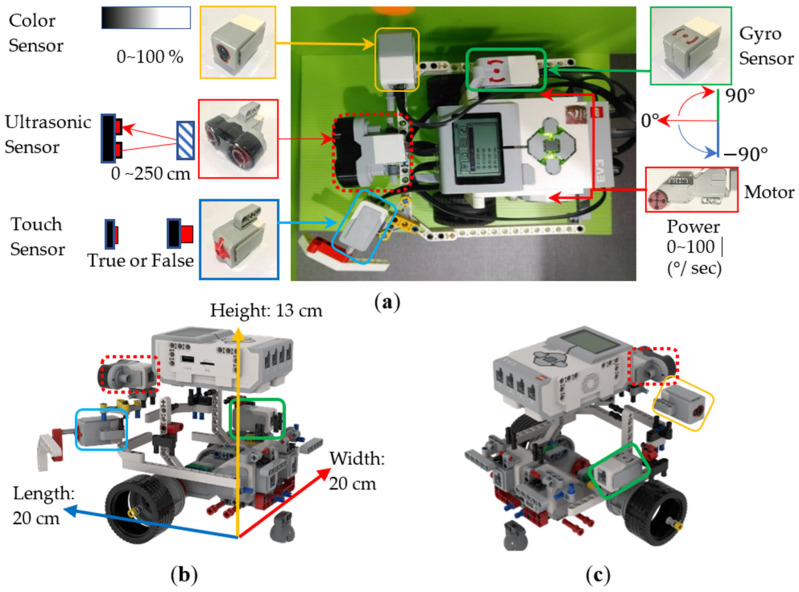
Reference example designed in the robot assembly process of the wall-following robotics experiment after the PDCA cyclic improvement process. (**a**) Top view. (**b**) Rear left view. (**c**) Rear right view.

Step 3: Plan and Do Robot Assembly

(Plan) Based on the experimental activities planned in Step 2, we planned a teaching example for students to assemble a wall-following robot. We used some mechanism components of LEGO EV3 and the Touch Sensor (TS), Ultrasonic Sensor (US), Color Sensor (CS), and Gyro Sensor (GS) shown in Table 4 to implement a two-wheeled mobile robot, and made some improvements. As shown in Figure 4, we implemented multiple wall-following robots to complete a teaching example. We initially only used three sensors: Touch Sensor (TS), Ultrasonic Sensor (US), and Color Sensor (CS). For example, as shown in Figure 4a, the TS was installed directly in front of the robot to detect the wall in front, while the US and CS were installed on both sides of the robot to detect whether the robot was close to the wall. But when the TS placed in front collides with the wall, unpredictable path deviation will occur. In addition, when the US placed on the side is too close to the wall (<3 cm), some error messages will often be generated. Therefore, we exchanged the assembly positions of US and TS so that the US could detect correctly. However, when the TS was assembled on the side of the robot, there will be a problem: it cannot collide with the wall vertically, resulting in insensitive response. Therefore, we designed an extension mechanism with a 45-degree angle (see Figure 4b) so that the TS is able to sense the touch of the wall more accurately. Finally, when the difficulty of the experimental activity was increased to moving along the wall in both directions, we added a GS to identify the direction of the robot. As shown in Figure 4c, we initially placed the GS on the side of the robot. It is known from experiments that if the GS is placed closer to the axis position when the robot turns, the sensed value will be more accurate. This enables the robot to judge the direction more accurately. Therefore, as shown in Figure 4d, the GS was finally placed at the center of the robot. After the PDCA cyclic improvement process, we generated a robot assembly reference example (see Figure 5) based on the robot shown in Figure 4c. Its dimensions were 20 × 20 × 13 cm in length, width, and height, and it consisted of 118 LEGO pieces. Based on the instructional design concept of Scaffolding Theory, the designed reference example did not place the GS at the center of the robot. The purpose was to stimulate students’ creativity in sensor assembly. There was only a reminder during classroom teaching that placing the GS at the center of the robot will allow it to sense a more accurate direction.

**Figure 6:**
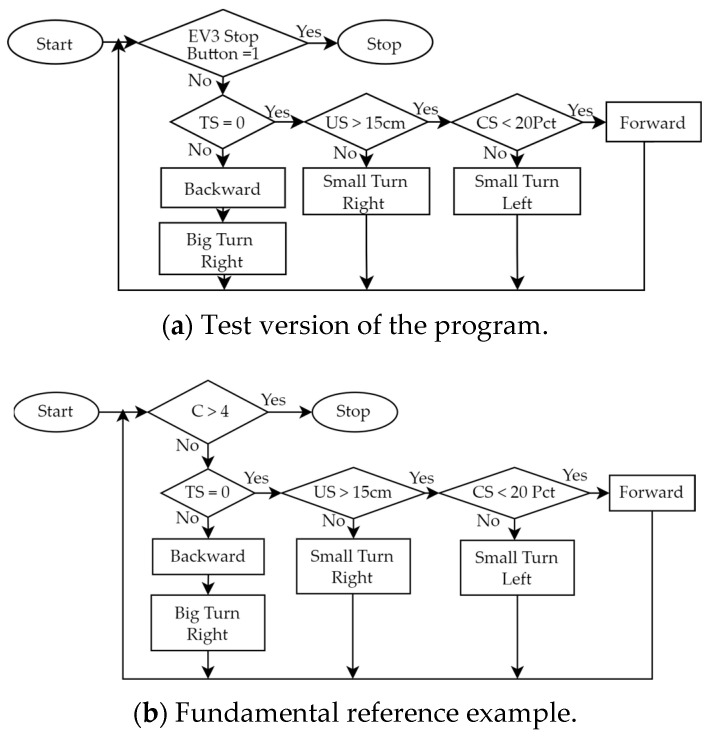
Two fundamental reference example programming flowcharts designed in the robot control of the wall-following robotics experiment before and after the PDCA cyclic improvement process.

**Figure 7:**
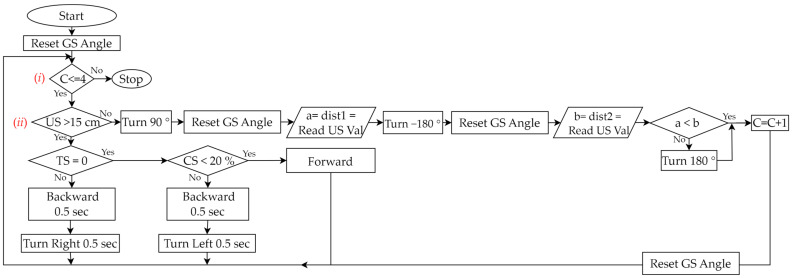
Advanced reference example designed in the robot control process of the wall-following robotics experiment. (i) Termination condition of the program. (ii) Judgment conditions based on sensor values.

Step 4: Plan and Do Robot Control

(Plan) Based on the experimental activities planned in Step 2 and the wall-following robot assembled in Step 3, we planned a teaching example that allows students to design programs to control the robot. We used the graphical programming language EV3-G and corresponding programming flowcharts, as shown in Table 5, to design the control program.
(Do) During the PDCA cyclic improvement process of using EV3-G programming blocks to build a teaching example of robot control, we found some problems and made some improvements. For example, although we used EV3-G programming blocks to design programs, we found that when the program structure is complex, using a general programming flowchart to illustrate the overall structure will make it easier for students to understand the program. Therefore, during the PDCA cyclic improvement process, we finally introduced the corresponding relationship between the programming blocks and general programming flowcharts in the classroom. Then, we initially only used “Loop” and “Switch (Selection)”, as shown in Table 5, to design a test version of the program, where “Loop” was used to allow all sensors to continuously detect, and “Selection” was used to process the movement of the robot based on the sensing value of each sensor. For example, based on the values sensed by the TS, US, and CS, we used judgments in the loop to control the movement of the robot. We initially used the infinite loop shown in Figure 6a, but the robot could not stop automatically when it reached the finish line. Therefore, we improved the design of the conditional loop, as shown in Figure 6b, where the counter (C) is used to determine the condition when the loop terminates, so that the robot can automatically stop after completing the task. Next, we developed a fundamental reference example, making it easy for beginners to get started. When the experimental activity changed from one-way wall-following to two-way wall-following, the control program had to be redesigned so that the program can allow the robot to complete both clockwise and counterclockwise wall-following tasks. We used three programming syntaxes, “Nested-Loop”, “Nested-Selection”, and “Variable”, as shown in Table 5. Figure 7 is a reference example of using these three new syntaxes to establish robot control for a wall-following robot. There were three main parts: (a) We designed a conditional loop C ≤ 4 as shown in Figure 7 (i) to terminate the program. (b) We used the judgments of the sensors’ values as shown in Figure 7 (ii) to execute the program. If (US > 15 cm and TS = 0 and CS < 20%) is true, the robot continued to move forward. If (US > 15 cm) is false, it means that the robot has reached the corner. Then, the robot turned to the right (90°) and left (−180°) to obtain the sensing values of the US and compared the two values to determine the next movement direction of the robot. Finally, the counter was added by 1 (C = C + 1). If (TS = 0 or CS < 20) is false, it means that the left or right side of the robot is too close to the wall, and the robot is allowed to retreat and rotate to the right or left for 0.5 s to correct the path. (c) Every time we used the GS, we had to reset GS to 0 degrees.

Step 5: Do and Check Students’ In-Class Performance

(Do) We performed the examples completed in Steps 3 and 4 and performed actual hands-on experiments using a physical robot.
(Check) Based on the in-class evaluation criteria planned in Step 2, as shown in Table 2, we checked students’ learning outcomes in experimental activities, robot assembly, robot control, and in-class performance.

Step 6: Act Based on Students’ In-Class Performance

(Act) Based on the students’ in-class performance, checked in Step 5, if the experimental activities, robot assembly, or robot control need to be improved, we will need to go back to Step 2, Step 3 or Step 4 to re-plan, respectively. See the following cases for examples: (a) In this example, we need to go back to Step 2 to improve the experimental activities. Some students were unable to complete the experiment when we did not provide reference examples at the beginning. But when we provided a fundamental reference example (moving clockwise along the wall), not only were all students able to complete the experiment, but many students could complete it early. Therefore, we returned to Step 2 to increase the difficulty of the experimental activity and provide a reference example in advance (such as moving along the wall in both directions). (b) In this example, we need to go back to Step 3 to improve the robot assembly. When we did not provide reference examples of robot assembly at the beginning, some students were unable to assemble the robot smoothly, and a small number of students even felt frustrated. Therefore, we returned to Step 3 to provide two videos of the step-by-step assembly of two-wheeled robots, a fundamental reference example of a simple two-wheeled mobile robot (see https://reurl.cc/xLgyN5), and an advanced reference example of a more complex wall-following robot (see https://reurl.cc/N4X3pm). The reference examples allow all students to easily master the fundamentals of robot assembly and to complete the experiment within the specified time. In addition, this improvement will encourage more students to use their creativity to assemble different types of robots in the classroom (see Section 4.1). (c) In this example, we need to go back to Step 4 to improve the robot control. Similarly, when we did not provide a reference example of robot control at the beginning, some students were not able to successfully complete the design of the control program. Therefore, we returned to Step 4 to provide two design programs of robot control, a fundamental reference example (see Figure 6 and https://reurl.cc/2zr1Ev), and an advanced reference example (see Figure 7 and https://reurl.cc/2zr1jn). The official free software download point is https://reurl.cc/WR36g5. These improvements will allow all students to easily master the fundamentals of control programming and to complete experiments within the specified time. Similarly, this improvement will encourage some students to be creative in the classroom and design programs that can complete experiments faster (see Section 4.2).

Step 7: Do and Check Students’ After-Class Reports

(Do) We performed the correction and analysis of reports submitted by students.
(Check) Based on the requirements of the after-class report planned in Step 2, as shown in Table 3, we checked students’ learning outcomes in experimental activities, robot assembly, robot control, and after-class reports.

Step 8: Act Based on Students’ After-Class Reports

(Act) Based on the students’ after-class reports, checked in Step 7, if the experimental activities, robot assembly, or robot control need to be improved, we need to go back to Step 2, Step 3, or Step 4 to re-plan. See the following cases for examples: (a) In this example, we need to go back to Step 2 to improve the experimental activities. When we only provided some rough topics of the after-class report at the beginning, some students’ reports had problems with content being in a confusing order or with unclear descriptions. Therefore, we went back to Step 2 to provide some requirements for the after-class report as shown in Table 3, which had clearer requirements for the four topics and sub-topics, so that students could write reports in sequence according to these requirements and learn how to write a specific experimental report. In addition, we found that these requirements can allow students to know which materials must be collected in order to write reports during the experimental process and to improve students’ concentration in the classroom. Moreover, we found from some students’ learning processes and feedback that they did not know how to debug and were hesitant to know where to start when they encountered problems. Therefore, we introduced the PDCA concepts in the classroom and let students use this cyclic step-by-step method to solve problems, and this adjustment was indeed reflected in students’ creative programming (see Section 4.1). (b) In this example, we need to go back to Step 3 to improve the robot assembly. From students’ learning experience and feedback, we found that when the GS was assembled at the center of the robot, it could improve the accuracy of the robot’s steering. Therefore, we returned to Step 3 to establish a reference example of robot assembly, as shown in Figure 4d. This improvement will encourage students to assemble different types of robots. (c) In this example, we need to go back to Step 4 to improve robot control. Similarly, from the students’ learning experiences and feedback, we found that when the program was complex, the overall graphical control program was confusing and difficult to understand. Therefore, we returned to Step 4 to add the function syntax “My Blocks” (“My Blocks” is equivalent to “Function” in program syntax), as shown in Table 5, to improve this problem.

## 4. Students’ Learning Outcomes

The PDCA design method proposed in this study was established after three stages of wall-following robotics experiments. Three groups of 42, 37, and 44 students participated in the experiment in these three stages, respectively. All students were first-year students in the Department of Electrical and Computer Engineering. They were all exposed to the robotics experiment using multiple sensors for the first time. The improvement in four key learning outcomes in these three stages is shown in Table 6. It can be seen from the table that: (1) The ratio of robots not colliding with walls increased from 0% to 50%. (2) The ratio of using gyro sensors increased from 2.38% to 100%. (3) The ratio of gyro sensors placed in the center improved from 2.38% to 56.82%. (4) The ratio of the time required for the robot to complete the wall-following experiment was less than the 75 s of the reference example, increased from 7.14% to 100%. It can be seen from the students’ learning outcomes in the third stage of the experiment that the proposed reference examples allowed students to quickly assemble robots and design programs to control the assembled robots. In addition, some students further stimulated their creativity to assemble different robots or control programs. Below are some of the students’ creative outcomes.

### 4.1. Students’ Creations in Robot Assembly

**Figure 8:**
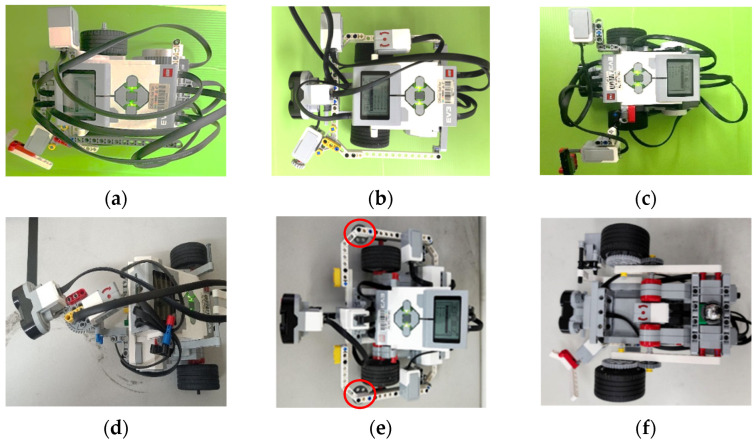
Students’ outcomes in the robot assembly of the wall-following robots. (**a**) Level A. (**b**) Level B. (**c**) Level C. (**d**) Level D: Robot can scan three directions. (**e**) Level D: Robot with auxiliary wheels. (**f**) Level D: GS was placed in the center of the robot.

The outcomes of six students in the assembly of the wall-following robot are shown in Figure 8. Based on the criteria shown in Table 2, the robots shown in Figure 8a–c are evaluated as Levels A, B, and C. Moreover, robots shown in Figure 8d–f are evaluated as Level D. Their creation is described as follows: As shown in Figure 8d, a medium motor is used to rotate the US so that it can scan in three directions. Due to the increased error tolerance of the mechanical structure, the robot can challenge any path. As shown in Figure 8e, the robot is equipped with two auxiliary wheels (indicated by red circles) to buffer the wall offset, so it has a faster speed than other students’ robots. As shown in Figure 8f, the GS is assembled directly at the center of the robot so that the robot can turn more accurately. Moreover, the distance between the robot’s two wheels (<15 cm) is shorter than the others, so the robot has a higher turning efficiency and fewer collisions.

### 4.2. Students’ Creations in Robot Control

**Figure 9:**
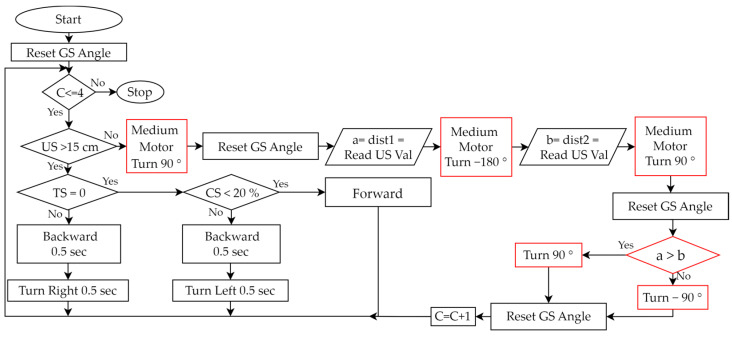
One creative programming flowchart designed by students.

**Figure 10:**
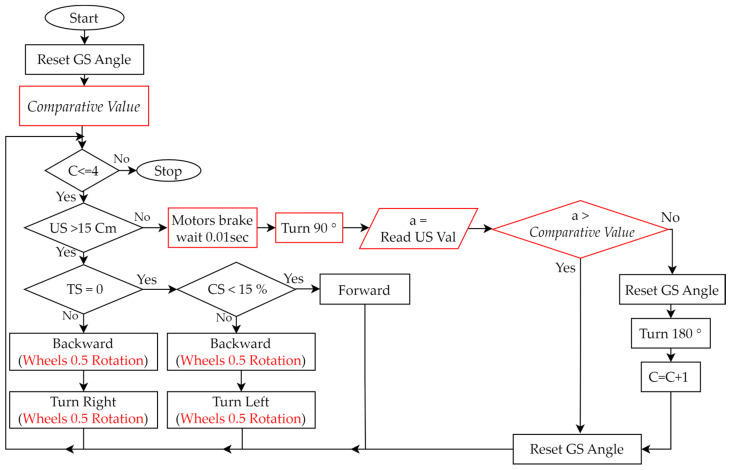
Another creative programming flowchart designed by students.

Two creative programs evaluated as Level D are described as follows: As shown in Figure 9, the programming flowchart of the provided advanced reference example has been significantly modified, with the major differences highlighted in red. Then, the assembled robot, as shown in Figure 8d, can be effectively controlled. When the robot is in the corners, its creative aspect is that a medium motor is controlled to rotate the US in two directions (90° and −180°), so there is no need to rotate the robot. Another creative programming flowchart is shown in Figure 10. This program uses a total of 36 programming blocks, which is 14 fewer than the programming blocks used in the reference example. At the same motor speed of 15 degrees/second, the student’s robot completed the wall-following task in 60 s, while the reference example required 75 s. The following paragraphs include the student’s clear description of his program and the PDCA process he used.

“In this experiment, my focus was on robot control, and I made modifications based on the teacher’s example program. The original program from the teacher measured distances on both sides when the robot was at the corner. Which direction to turn is decided based on these two measured values. This mode had a high accuracy rate, but the completion speed was relatively slow. Therefore, I changed it to detect only one side (left or right). Once the robot is at the corner and the US value is less than 12 ± 3cm, the robot would proceed the decision of direction. This approach reduces the total number of the US measurement, but the drawback is that it works well only in relatively regular road conditions. It may encounter errors if the road width changes too dramatically or if the road conditions are more complex. I anticipate that adding some confirmed values might address this problem”.

In the after-class report, this student reported that he applied the following PDCA concepts to design his program.

Step 1: (Plan) Compared to the reference example, I planned to reduce the number of measurements using the US at corners.
Step 2: (Do) I reduced the number of measurements at the corners from two in the reference example to one. In addition, I needed to determine a comparative value at 12 ± 3 cm for the US.
Step 3: (Check) The total number of measurements using the US at corners was reduced from six to three, so the execution time of this method was less than that of the reference example. However, when the field changes significantly, some problems may arise.
Step 4: (Act) Based on multiple tests, the comparative value (12 ± 3 cm) used was 15 cm.

## 5. Conclusions

In research on improving student learning outcomes, there is no complete and interoperable method for designing robotics experiments. Hence, a student-oriented approach based on PDCA to design robotics experiments is proposed in this study. Firstly, the teaching experience and students’ learning outcomes in the experimental design of wall-following robots are used to describe the proposed PDCA method. This experiment also contributes to teaching students about the fundamental applications of multi-sensor fusion technology. The proposed experimental design method has teaching scalability. Some illustration examples of the designed process can allow teachers to use the proposed method easily to design any robotics experiments with different robot platforms and different programming languages. Secondly, the proposed PDCA method is a student-oriented method that can enhance students’ learning outcomes. The proposed reference examples allow students to quickly assemble two-wheeled mobile robots with four different sensors and design programs to control these assembled robots. In addition, the proposed in-class evaluation criteria stimulate students’ creativity to assemble different wall-following robots or design different programs to achieve this experiment. Three groups of 42, 37, and 44 students participated in the wall-following experiment in three stages, respectively. The ratios of students using the gyro sensor in these three stages were 1/42 = 2.38%, 10/37 = 27.03%, and 44/44 = 100%, respectively. The ratios of placing the gyro sensor in the center of the robot were 1/42 = 2.38%, 5/37 = 13.51%, and 25/44 = 56.82%, respectively. The ratios of the time required for the robot to complete the wall-following experiment in less time than the teaching example were 3/42 = 7.14%, 26/37 = 70.27%, and 44/44 = 100%, respectively. The comparison of learning outcomes in the three stages can illustrate that the proposed method can indeed improve students’ learning outcomes and stimulate their active learning and creativity. Finally, the proposed method develops students’ report-writing skills. This is rarely mentioned in other works in the robot education literature. The after-class reports written by the students following the requirements designed by the proposed method can allow teachers to understand the students’ learning outcomes of robot assembly and robot control in the classroom as well as the students’ feedback after class. According to the analysis of after-class reports by 44 students (35 male and 9 female) in the third stage of the experiment, 100% of the students felt that the designed experiment could help them understand how to use multiple sensors to effectively assemble wall-following robots, and how to design programs to control the robots. In addition, the designed course could also help them to improve their problem-solving and report-writing abilities. The numerical evidence and satisfaction feedback supports the proposed PDCA-based robotics experimental design method as a way to help students quickly understand and immediately implement a hands-on robotics experiment. This enables them to achieve good learning outcomes within a limited period. In future work, we plan to study how to effectively apply the proposed method to design control experiments for more complex robots (such as SCARA four-axis robotic manipulators or six-axis robotic manipulators).

## Figures and Tables

**Table 1:** Two different types of software and hardware used in various works in the robotics course-related literature.

	Software	Text-Based Programming Language	Graphical Programming Language
Hardware			
Arduino-series Platform		Arduino, Arduino C [14,15] FRDM-KL05Z, C [16] TCLab Arduino Kit, MATLAB or Python [17]	Arduino, LabView and Scratch [18]
LEGO-series Platform		LEGO EV3, MATLAB [19,20]	LEGO EV3, Block-based Programming [21] LEGO EV3, EV3-G [22] LEGO EV3, LabView [23] LEGO EV3, Scratch [24] LEGO WeDo, Scratch [25]

**Table 2:** In-class evaluation criteria obtained by the proposed PDCA-based design method.

Completion Mode	Level
Use the given fundamental version of the reference example.	Level A
Use the given advanced version of the reference example.	Level B
Significantly modify the given fundamental version of the reference example.	Level C
Significantly modify the given advanced version of the reference example, or make it more concise or creative.	Level D

**Table 3:** Requirements of after-class report obtained by the proposed PDCA-based design method.

Topics Requirements
1. Experimental Objectives 1.1. Course Objectives 1.2. Other Course Objectives (Optional)
2. Experimental Tasks and Principles 2.1. Experimental Tasks and Principles 2.2. Other Experimental Tasks or Principles (Optional)
3. Experimental Results 3.1. Robot Assembly (the design results must be described step by step, and the content must have clear front, rear, top, and side photos of the robot, and the installation locations of the sensors must be marked) 3.2. Robot Control (the design results must be described step by step, and the content must include a flowchart of the control program and a description of the programming concept)
4. Learning Experience and Feedback 4.1. Robot Assembly (the content must include the experience of installing sensors or assembling the robot) 4.2. Robot Control (the content must include the experience of programming) 4.3. After-class Report (the content must include the experience of writing the after-class report)

**Table 4:** Photos of sensors, programming blocks, and mechanism components with sensors.

Name	Touch Sensor	Ultrasonic Sensor	Color Sensor	Gyro Sensor
Sensors	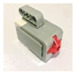	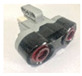	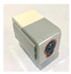	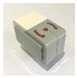
EV3-G Programming Blocks	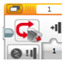	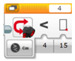	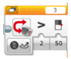	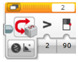
Mechanism Components with Sensors	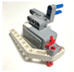	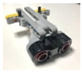	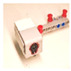	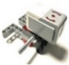

**Table 5:** Names of the programming syntax of the EV3-G programming blocks used in the wall-following robotics experiment and the corresponding general programming flowcharts.

Name	Loop	Switch (Selection)	Nested-Loop	Nested- Selection	Variable	My Blocks (Function)
EV3-G Programming Block	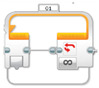	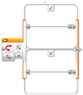	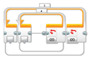	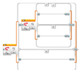	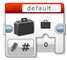	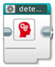
Programming Flowchart	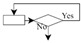	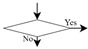	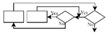	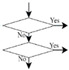	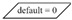	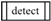

**Table 6:** Learning results of three groups of students (42, 37, and 44 students) in the wall-following robotics experiment in the three stages of the proposed PDCA method.

	First Stage	Second Stage	Third Stage
Robots did not collide with walls	0/42 = 0%	5/37 = 13.51%	22/44 = 50%
Use of the gyro sensor	1/42 = 2.38%	10/37 = 27.03%	44/44 = 100%
Gyro sensor placed in the center	1/42 = 2.38%	5/37 = 13.51%	25/44 = 56.82%
Less time than reference example	3/42 = 7.14%	26/37 = 70.27%	44/44 = 100%

## Data Availability

Data are contained within the article.
